# Incidence and characteristics of Traumatic Tympanic Membrane perforation

**DOI:** 10.12669/pjms.345.15300

**Published:** 2018

**Authors:** Fazal I. Wahid, Sajid Rashid Nagra

**Affiliations:** 1 Dr. Fazal I. Wahid, FCPS. Department of E.N.T, Head & Neck Surgery, Medical Teaching Institute (MTI), Lady Reading Hospital (LRH), Peshawar, Khyber Pakhtunkhwa, Pakistan; 2 Dr. Sajid Rashid Nagra, FCPS. Department of E.N.T, Rai Medical College, Sargodha, Pakistan

**Keywords:** Membrane, Perforation, Slap, Tympanic Trauma

## Abstract

**Objective:**

To determine incidence and characteristics of traumatic Tympanic Membrane perforation in a tertiary care hospital.

**Methods:**

This descriptive cross sectional study was conducted at the Department of ENT, MTI/LRH, Peshawar from August to December 2017. After well informed consent a detailed history was taken; thorough examination of ENT was carried out. Pure Tone Audiometry (PTA) of all included patients was performed by same senior audiometrician. The data were collected on predesigned proforma and were analyzed using SPSS (16 version

**Results:**

This study included 48 patients; Male 34, Female 14 with Male: Female ratio of 2.24:1. The mean age was 26.75 ± SD 9.88 Years. Majority of the patients (24, 50%) were in 3rd decade of life. The commonest cause of perforation of tympanic membrane was slap (35, 72.9%). Perforation of the Tympanic membrane was found more on left side (24, 50%), involving anterio-inferior site (18, 37.5%), while medium sized perforation was frequent finding (27, 56.2%). Single perforation was commonest observation (45, 93.8%), conductive hearing loss was the most common finding (38, 79.2%), and mild degree hearing loss was commonest (24, 50%).

**Conclusion:**

Traumatic tympanic membrane perforation is common in young male. Slap was the commonest cause of traumatic perforation affecting left ear more than right ear. Inferior quadrant of the tympanic membrane was commonly involved and mild degree and conductive type of hearing loss was common.

## INTRODUCTION

Human Tympanic membrane (TM) is a thin semi-translucent, pearly white membrane that separates middle ear from external ear. It lies obliquely at the medial end of external auditory meatus forming major part of lateral wall of middle ear cavity. It is approximately 10 mm and 5 mm in vertical and horizontal diameter respectively. It is made of three layers; outer epithelial, middle fibrous layer composed of circular and radial fibers and inner layer is mucosal.^1^ The main function of TM is amplification and transmission of sound waves from external auditory canal through ossicular chain to oval window and vestibular ramp due to its vibratory characteristic. It also protects middle ear cleft.^2^ Tympanic membrane perforation is a pathological condition as old as the human species.^3^ The incidence of traumatic TM perforation is reported in literature at 6.80/1000 persons.^4^ The traumatic TM perforation is classified according to duration, acute (< 3 months) and chronic (> 3 months) and by the presence or absence of otorrhea in wet and dry perforation respectively.^5^ The perforation can also be classified according the size. The two commonly used method of calculating size are:

1. Percentage of perforation=Perforation/ Total area of TM x 100 %(In this method perforation is measures in pixels). Thus perforation can be classified into Group-I (Small)- area in range of 0-8 mm^2^ Group-II (Medium)- area in range of 8.1 -30 mm^2^, Group-III (large) – area in range ≥30.1 mm^2^.^6^
2. In this method perforation size can be calculated using equation; Percentage of perforation= Area of perforation/ Total area of perforation x100. Thus perforation can be categorized as Small (area involved is ≤25% or one quadrant), Medium (area involved is 25% -50% or two quadrants), and Large (area involved is 50%-75% or more than two quadrants).^2^

The causes of traumatic TM perforation include sudden increase in ear pressure due to slapping, blast, forceful syringing and caloric tests, faulty technique of ear cleaning or extracting FB, probing, accidents, travelling in a non-pressurized air craft or sudden fluid compression while diving.^1^ The incidence of traumatic TM perforation is on rise globally due to interpersonal violence, more industrialization and weapon misuse. Traumatic TM perforation can manifest as sudden severe pain, hearing impairment, bleeding, tinnitus, dizziness, perilymph fistula and facial nerve injury.^7^ Traumatic TM perforation may result into suppurative otitis media and sensorineural hearing loss if not treated in time. The treatment modality of traumatic TM perforation include non-surgical and surgical. It has 78. % spontaneous healing rate while in some study it is 90%.^2^,^4^ If spontaneous healing fails after six months then myringoplasty or tympanoplasty are carried out if it is also associated with significant conductive hearing loss.^2^

Traumatic TM perforation is very common clinical condition presented to otorhinolaryngologist. A significant number of patients are received by our department, so this study is aimed keeping in view the following points:

1. To quantify the burden of the disease as such study is not carried out in our set up.
2. To know the details of this condition to formulate any preventive measures.

## METHODS

This descriptive cross sectional study was conducted at the Department of ENT, Head and Neck surgery, Medical Teaching Institute, Lady Reading Hospital Peshawar after getting approval from hospital Ethical board from August 2017 to December 2017 (5 months).

### Inclusion criteria were

1. Patients of all age and both gender sustaining traumatic tympanic membrane perforation.
2. Patients willing to be included in study.

### Exclusion Criteria were

1. Non-traumatic tympanic membrane perforation.
2. Traumatic perforation with severe head injuries or patients with poly trauma.

After administering well informed consent to the patients a detailed history was taken; thorough examination of ENT specifically otoscopic examination of ears and systems review was carried out. Pure Tone Audiometry (PTA) of all included patients was performed by same senior audiometrician. The data were collected on predesigned proforma and were analyzed using SPSS (16 version).

## RESULTS

This study included 48 patients; Male 34, Female 14 with Male: Female ratio of 2.24:1. The age of the patients ranged from minimum of 6 to maximum 55 years, with mean of 26.75 ± SD 9.88 Years. The median and mode of the age were 25 and 35 years respectively. Majority of the patients (24, 50%) were in 3rd decade of life followed by 4^th^ and 2^nd^ decades (10, 20%, 9, 18.8%) respectively (Table-I). The commonest cause of perforation of tympanic membrane was slap (35, 72.9%), followed by blast injury (4, 8.3%) (Table-II). Perforation of the Tympanic membrane was found more on left side (24, 50%), involving anterio-inferior site (18, 37.5%) more than other sites, while medium sized perforation was frequent finding (27, 56.2%) (Table-III). Most of the patients (32, 66.7%) presented to ENT OPD within a week time of receiving TM perforation having dry ear (44, 91.7%) and single perforation was commonest observation (45, 93.8%). On performing pure tone audiometry conductive hearing loss was the most common finding (38, 79.2%), while mild degree hearing loss was commonest (24, 50%) finding (Table-IV).

**Table-I:** Age distribution of patients in decades (n=48).

Age in Years	Frequency	Percent	Valid Percent	Cumulative Percent
<10	2	4.2	4.2	4.2
10- 19	9	18.8	18.8	22.9
20 - 29	24	50.0	50.0	72.9
30 - 39	10	20.8	20.8	93.8
40 - 49	2	4.2	4.2	97.9
≥ 51	1	2.1	2.1	100.0

Total	48	100.0	100.0	

**Table-II:** Cause of the TM perforation (n=48).

Cause of the TM perforation	Frequency	Percent	Valid Percent	Cumulative Percent
Slap	35	72.9	72.9	72.9
Blast	4	8.3	8.3	81.2
FAI	2	4.2	4.2	85.4
Stick	3	6.2	6.2	91.7
Suction/ Probing/syringing	3	6.2	6.2	97.9
Road Traffic Accident	1	2.1	2.1	100.0

Total	48	100.0	100.0	

**Table-III:** Distribution of TM perforation by side, site and size (n=48).

*Side-wise distribution of TM perforation*			

*Side of Ear*	*Frequency*	*Percent*	*Valid Percent*

Right ear	20	41.7	41.7
Left ear	24	50.0	50.0
Bilateral	4	8.3	8.3

Total	48	100.0	100.0

*Site-wise distribution of TM perforation*			

*Site of Perforation*	*Frequency*	*Percent*	*Valid Percent*

Anterio-superior	4	8.3	8.3
Anterio-inferior	18	37.5	37.5
Posterio-superior	14	29.2	29.2
Posterio-inferior	12	25.0	25.0

Total	48	100.0	100.0

*Size-wise distribution of TM perforation*			

*Size of Perforation*	*Frequency*	*Percent*	*Valid Percent*

Small	20	41.7	41.7
Medium	27	56.2	56.2
Large	1	2.1	2.1

Total	48	100.0	100.0

**Table-IV:** Duration, status, number of perforations and hearing loss. (n=48).

*Duration of perforation*			

*Duration of Perforation*	*Frequency*	*Percent*	*Valid Percent*

< a week	32	66.7	66.7
< a month	13	27.1	27.1
> a month	3	6.2	6.2

Total	48	100.0	100.0

*Status of the ear*			

*Status of Perforation*	*Frequency*	*Percent*	*Valid Percent*

Dry	44	91.7	91.7
Wet	4	8.3	8.3

Total	48	100.0	100.0

*Number of perforation*			

*Number of Perforation*	*Frequency*	*Percent*	*Valid Percent*

Single	45	93.8	93.8
Multiple	3	6.2	6.2

Total	48	100.0	100.0

*Pure tone audiometry finding*			

*PTA finding*	*Frequency*	*Percent*	*Valid Percent*

Conductive Hearing Loss	38	79.2	79.2
Sensorineural Hearing Loss	9	18.8	18.8
Mixed Hearing Loss	1	2.1	2.1

Total	48	100.0	100.0

*Grades of hearing Impairment*			

*Grades of hearing Impairment*	*Frequency*	*Percent*	*Valid Percent*

Mild Hearing Loss	24	50.0	50.0
Moderate Hearing Loss	17	35.4	35.4
Severe Hearing Loss	7	14.6	14.6

Total	48	100.0	100.0

## DISCUSSION

Perforation of the tympanic membrane (TM) due to trauma is a common occurrence across the globe due to human violence. The Pathophysiology of traumatic TM perforation is sudden rise in the air pressure of the external auditory canal which leads to rupture of the thinnest part of the membrane to the extent depending upon the amount of change of pressure. In this study total 48 cases were included with male preponderance 2.24: 1, which is in accordance to study from Bangladesh (Rabbani and collegues) included 70 patients with male predominance and Dawood and colleges from Iraq studied 62 patient with traumatic perforation with male gender dominance.^1^, ^2^ The age of the patient ranged from 6 to 55 years with mean age and SD of 26.75 ± SD 9.88 years in this study, that is close to the results of Onotai from Nigeria where age range was 2-70 years with a mean age of 35.2 years ± 6.3. This is also supported by another study from Nigeria by Chukuezi who found that the age ranged from 12–43 years with average of 26.3 ± 5 years.^3^,^8^ Majority of the patients (24, 50%) were in 3rd decade of life in this study. This 3^rd^ decade of life is also commonly involved in studies of Rabbani, Dawood and Onotai (65.7%, 40.30%, and 28.57%), because the people in this decade are more active and prone to violence. The commonest cause of perforation of tympanic membrane was slap (35, 72.9%), followed by blast injury (4, 8.3%) in this study, that is in conformity with results of Rabbani SMG, Onotai LO, Wani A, Saimanohar S, Sarojamma Q and Perera MC where slapping accounts 88.5%, 42.86%, 69.42%, 86.67%,50% and 75% respectively.^1^,^3^-^5^,^9^,^10^ However it is contrary to study of Dawood from Iraq where blast injury was common 43. 50% than slap 25.80% leading to TM perforation, because blasts are common in Iraq which is in a state of war for last so many years.^2^ In our study perforation of the Tympanic membrane was found more on left side (24, 50%), involving anterio-inferior site (18, 37.5%) more than other sites, while medium sized perforation was frequent finding (27, 56.2%). These results are in agreement with results of Rabbani with left side and inferior quadrant of TM perforation accounts 85.0%, Onotai LO study’s left ear involvement was 52.29%, inferior quadrant perforation was 42.86%, Wani study’s left side involvement was 71.15% and medium size perforation was 63.14%.^1^,^3^,^4^, Similarly Sarojamma also reported that left ear was affected in 66% and posterio-inferior quadrant of TM was perforated in 58%.^9^ Similar result is also reported by Ravi, where lower half of the TM was perforated in 96%.^11^

The result of this study varies from Dawood’s study, where small size perforation was found in 51.5%, and posterior quadrant TM perforation found in 50% and that of Mazumder’s study where small size perforation was more common (60%) and postero-inferior quadrant was involved in 61.90% patients.^2^,^12^ The probable explanation of left side involvement is that right handed person tends to slap the victim facing to him over the left ear. The air pressure change produced in the external auditory canal by slap is sufficient to cause medium size perforation in the tympanic membrane, as unveiled from the results of this study that commonest cause of perforation of tympanic membrane was slap (72.9%), while medium sized perforation was commonest perforation (56.2%). Inferior quadrant of the TM has more range of mobility as compared to upper half which is supported by chain of ossicles, that’s why perforation was commonly observed in lower half of the tympanic membrane. In current study most of the patients (32, 66.7%) presented to ENT OPD within a week time of receiving TM perforation. The ears were infection free in 44 cases (91.7%) and single perforation was commonest observation (45, 93.8%), that is in accordance with result of Wani who found single perforation in tympanic membrane in 92.3% patients while multiple perforations were found in 7.7% patients.^4^ Similarly in Park study the commonest finding was single perforation in TM.^7^ Majority of patients consulted ENT OPD after receiving trauma to ear except in cases where trauma was not resulting into any significant symptom or patient belonged to a far flung area

On performing pure tone audiometry conductive hearing loss was the most common finding (38, 79.2%), while mild degree hearing loss was commonest (24, 50%) finding. This is comparable to results of Wani who found that majority of patients (62%) presented with conductive hearing loss of mild degree hearing impairement.^4^ Similar findings were also noted by Sarojamma and Ravi where majority of patients (62% and 60%) presented with conductive hearing loss in the range of 26- 35dB (mild degree conductive hearing loss).^9^, ^11^ The result of this study in terms of degree of hearing loss was also consistent with Raj, Rehman, Singh and Khan’s study where mild degree hearing loss was reported 66.66%, 49.45%, 66.66% and 62% rrespectively.^13^-^16^

## CONCLUSION

Traumatic tympanic membrane perforation is common in young male especially in 3^rd^ decade of life. Slap was the commonest cause of traumatic perforation affecting left ear more than right ear. Inferior quadrant of the tympanic membrane was commonly involved and mild degree and conductive type of hearing loss was common.

### Author`s Contribution

**WFI** conceived, designed and prepared the manuscript.

**NSR** did statistical analysis & proof reading of manuscript.
